# Chronic nonbacterial osteomyelitis in childhood: prospective follow-up during the first year of anti-inflammatory treatment

**DOI:** 10.1186/ar2992

**Published:** 2010-04-30

**Authors:** Christine Beck, Henner Morbach, Meinrad Beer, Martin Stenzel, Dennis Tappe, Stefan Gattenlöhner, Ulrich Hofmann, Peter Raab, Hermann J Girschick

**Affiliations:** 1 Children's Hospital, Section of Paediatric Rheumatology, Osteology, Immunology and Infectious Diseases, University of Würzburg, Josef Schneider Straße 2, 97080 Würzburg, Germany; 2 Institute of Radiology, Department of Pediatric Radiology, University of Würzburg, Josef Schneider Straße 2, 97080 Würzburg, Germany; 3 University of Jena, Institute of Radiology, Department of Pediatric Radiology, Bachstraße 18, 07743 Jena, Germany; 4 Institutes of Hygiene and Microbiology, University of Würzburg, Josef Schneider Straße 2, 97080 Würzburg, Germany; 5 Institute of Pathology, University of Würzburg, Josef Schneider Straße 2, 97080 Würzburg, Germany; 6 Department of Internal Medicine I, University of Würzburg, Josef Schneider Straße 2, 97080 Würzburg, Germany; 7 Department of Orthopedics, Section of Pediatric Orthopedics, Koenig-Ludwig-Haus, Brettreichstraße 11, 97074 Würzburg, Germany; 8 Vivantes Children's Hospital, Pediatric Rheumatology, Immunology and Infectious diseases, Landsberger Allee 49, 10249 Berlin-Friedrichshain, Germany

## Abstract

**Introduction:**

Chronic nonbacterial osteomyelitis (CNO) is an inflammatory disorder of unknown etiology. In children and adolescents CNO predominantly affects the metaphyses of the long bones, but lesions can occur at any site of the skeleton. Prospectively followed cohorts using a standardized protocol in diagnosis and treatment have rarely been reported.

**Methods:**

Thirty-seven children diagnosed with CNO were treated with naproxen continuously for the first 6 months. If assessment at that time revealed progressive disease or no further improvement, sulfasalazine and short-term corticosteroids were added. The aims of our short-term follow-up study were to describe treatment response in detail and to identify potential risk factors for an unfavorable outcome.

**Results:**

Naproxen treatment was highly effective in general, inducing a symptom-free status in 43% of our patients after 6 months. However, four nonsteroidal anti-inflammatory drug (NSAID) partial-responders were additionally treated with sulfasalazine and short-term corticosteroids. The total number of clinical detectable lesions was significantly reduced. Mean disease activity estimated by the patient/physician and the physical aspect of health-related quality of life including functional ability (global assessment/childhood health assessment questionnaire and childhood health assessment questionnaire) and pain improved significantly. Forty-one percent of our patients showed radiological relapses, but 67% of them were clinically silent.

**Conclusions:**

Most children show a favorable clinical course in the first year of anti-inflammatory treatment with NSAIDs. Relapses and new radiological lesions can occur at any time and at any site in the skeleton but may not be clinically symptomatic. Whole-body magnetic resonance imaging proved to be very sensitive for initial and follow-up diagnostics.

## Introduction

Chronic nonbacterial osteomyelitis (CNO) is an inflammatory, non-infectious disorder of the musculoskeletal system of unknown etiology. Both single and multiple lesions and recurrence have been described [1-3]. In children and adolescents CNO predominantly affects the metaphyses of the long bones, but lesions can occur at any site of the skeleton. Other organs including the skin, eyes, gastrointestinal tract and lungs can also be affected by inflammation [4-7]. Chronic recurrent multifocal osteomyelitis (CRMO) is considered the pediatric form of the SAPHO syndrome (synovitis, acne, pustulosis, hyperostosis and osteitis) and is the most severe form of CNO [8,9]. Histological bone lesions in unifocal and multifocal CNO, as well as in SAPHO syndrome, show similar inflammatory features [10,11].

There have been attempts to classify patients into defined groups (unifocal nonrecurrent, unifocal recurrent, multifocal nonrecurrent, multifocal recurrent) in order to set up diagnostic criteria and to find prognostic indicators [12,13]. CNO is diagnosed by exclusion of differential diagnoses such as malignancy, benign tumorous bone lesions, bacterial osteomyelitis, bone bruise or fracture, osteonecrosis and osteopetrosis. Currently, diagnosis is made by the clinical picture, laboratory data, radiological and magnetic resonance imaging (MRI) studies, technetium bone scan, and microbial and histopathologic analysis in a multidisciplinary approach. Until now no standardized diagnostic criteria and therapeutic guidelines or standards exist. Furthermore, there are no generally accepted treatment protocols available.

Nonsteroidal anti-inflammatory drugs (NSAIDs) have been recommended as a first-line therapy and seem to be safe and effective. Disease-modifying anti-rheumatic drugs (DMARDs), steroids, bisphosphonates and TNF blockers have also been used in severe disease manifestations, frequent relapses and associated inflammatory diseases [12,14-21]. Of note, bisphosphonates have also been used recently as first-line therapy [14,15,20]. Since treatment response using NSAIDs so far has been described only in retrospectively evaluated cohorts, we have initiated a prospective 5-year follow-up study. In the present article we describe the follow-up in the first year.

The aims of our short-term follow-up study were to evaluate NSAID treatment in the first year by describing clinical and whole-body (WB) MRI data and to identify risk factors for an unfavorable course of disease. NSAID treatment was expanded after 6 months using sulfasalazine and short-term steroids if treatment efficacy seemed limited. This treatment step was based on previous experience [12,13,16,18]. Special emphasis was placed on evaluation of a clinical scoring system, laboratory analysis and sequential WB-MRI in order to find diagnostic and prognostic indicators that could be helpful in the clinical management and future evaluation of different treatment strategies.

## Materials and methods

Thirty-seven children (24 girls, 13 boys) diagnosed with CNO were included who were newly diagnosed and who had not received any anti-inflammatory or antibiotic therapy. The disease was assessed using a clinical score, initial diagnostic biopsy, laboratory tests and multiple imaging including WB-MRI at 0, 3, 6 and 12 months. The CNO core set of outcome variables is composed of the following five measures: erythrocyte sedimentation rate (ESR), number of radiological lesions, severity of disease estimated by the physician, severity of disease estimated by the patient or parent, and the childhood health assessment questionnaire (CHAQ). The definition of improvement was as follows: for the PedCNO30 (PedCNO50, PedCNO70) score, at least 30% (50%, 70%) improvement in at least three out of five core set variables, with no more than one of the remaining variables deteriorating by more than 30% (50%, 70%).

Treatment with naproxen 15 mg/kg/day started at the time of diagnosis/biopsy and continued throughout 12 months. In case of insufficient response after 6 months, sulfasalazine was added at 20 mg/kg/day as a DMARD [14,19,20]. In addition, oral glucocorticoids were administered for 1 week at 2 mg prednisone/kg/day, followed by another week of tapering and discontinuation (for more detailed description see Additional file 1).

The study was approved by the local ethics committee. Signed informed consent was obtained from the patients' parents and from adolescent patients.

## Results

### Clinical features

#### Osteomyelitis

There was a mean delay of 5 months in making the diagnosis after the first symptoms had appeared. In all patients the disease onset was before 18 years of life, ranging from 25 months to 16 years of age with a mean age of 11.0 years. Eight patients showed a unifocal lesion, and 29 had multifocal lesions - 27 at the time of diagnosis, and two initially presented as a unifocal lesion but developed additional bone lesions during the first year of the disease. All together, 184 clinical foci (pain, functional impairment or swelling) were detected over 1 year (initial, 79 foci; after 1 month, 38 foci; after 3 months, 27 foci; after 6 months, 21 foci; and after 12 months, 19 foci) - resulting in a mean of 1.0 (2.1 at time of diagnosis, 1.1 after 1 month, 0.8 after 3 months, 0.6 after 6 months, and 0.5 after 1 year of treatment) per patient, showing a significantly lower number in follow-ups (analysis of variance (ANOVA), *P *< 0.05). The number of clinical lesions in the thorax, spine, pelvis and extremities were significantly less in the follow-ups of months 3, 6, and 12, whereas the head involvement remained unchanged (n = 1) on a low level (ANOVA, *P *< 0.05). The head was clinically involved in 2.7%, the extremities in 53.3%, the thorax in 19.6%, the spine in 6.5% and the pelvis in 17.9% of patients (Table 1).

**Table 1:** Course of disease: clinically and radiologically identified lesions located in all body regions

Location	0 months	3 months	6 months	12 months	Total in first year
Clinical lesions	2.1	0.8	0.6	0.5	1.0
Mean					
Absolute number	79	27^a^	21^a^	19^a^	184
Head	1 (1.3)	1 (3.7)	1 (4.8)	1 (5.3)	4 (2.7)
Extremities	38 (48.1)	15^a ^(55.6)	14^a ^(66.7)	10^a ^(52.6)	77 (53.3)
Thorax	15 (19.0)	6^a ^(22.2)	2^a ^(9.5)	4^a ^(21.1)	27 (19.6)
Spine	7 (8.9)	1^a ^(3.7)	2^a ^(9.5)	1^a ^(5.3)	11 (6.5)
Pelvis	18 (22.8)	4^a ^(14.8)	2^a ^(9.5)	3^a ^(15.8)	27 (17.9)
Radiological lesions					
Mean	5.0	3.7	2.5	2.2	3.4
Absolute number	184	121	89^a^	81^a^	475
Head	1 (0.5)	1 (0.8)	1 (1.1)	1 (1.2)	4 (0.8)
Extremities	91 (49.5)	78 (64.5)	66 (74.1)	63 (77.7)	298 (62.7)
Thorax	19 (10.3)	11 (9.1)	10 (11.2)	6^a ^(7.4)	46 (9.7)
Spine	27 (14.7)	9 (7.4)	2^a ^(2.2)	2^a ^(2.5)	40 (8.4)
Pelvis	46 (25.0)	22 (18.2)	10^a ^(11.2)	9^a ^(11.1)	87 (18.3)

Results presented as absolute numbers (%). Statistical analysis performed using analysis of variance. Head involvement was noted in one patient, where the os zygomaticum was affected.^a^*P *< 0.05 versus month 0.

At the time of diagnosis, 22% of patients complained about morning stiffness lasting 2 to 60 minutes (mean 12.5 minutes), 67% showed a relieving posture, 37% presented local bone/tissue swelling and 26% presented asymmetry of the extremities or thorax. Local pain in the affected bones was the leading symptom in 74% and was recorded with a mean score of 4.4 (unifocal 3.4, multifocal 4.8) using a 10 cm visual analog scale (VAS). Severity of disease was estimated by the parents/patient as 5.0 (unifocal, 4.5; multifocal, 5.1) on the VAS, and by the examiner as 4.7 (unifocal, 3.8; multifocal, 5.0). The global assessment/CHAQ was estimated as 3.8 (unifocal, 2.0; multifocal, 4.5), and the CHAQ score was 0.7 (unifocal, 0.1; multifocal, 1.0) at initial presentation.

#### Arthritis

Initially 14/37 (38%) patients were diagnosed with arthritis of joints adjacent to the lesion by physical examination and/or MRI. We did not diagnose arthritis in patients who were initially arthritis free during the first year. After 3 months arthritis was still present in all of these 14 patients, after 6 months in seven patients, and after 12 months three patients were still affected by arthritis.

#### Associated diseases

In 24% (9/37) of the patients, CNO-associated diseases were present.

Skin lesions such as palmoplantar pustulosis (3/6), acne conglobata (2/6) or psoriasis/psoriatic afflictions of nails (1/6) were present in 17% of patients (n = 6). In general, skin lesions tended to improve during the first year using emollients. Four out of these six patients had complete remission of their skin lesion.

Chronic inflammatory bowel disease was diagnosed in one patient (3%) initially. He was affected by Crohn's disease and showed multifocal and chronic inflammatory bone lesions. The patient needed a multimodal anti-inflammatory, immunosuppressant and immunomodulating therapy (naproxen, sulfasalazine, prednisone/budesonid, azathioprine) focusing on both clinical entities from the beginning because of many osteoinflammatory lesions (radiological, 19 lesions; clinical, five lesions) and severe bowel disease. In this patient, intestinal symptoms and signs of chronic bowel inflammation soon improved significantly, reaching clinical remission after 3 months. CNO disease activity, however, could not be brought into remission in parallel. Relapses, radiological signs of active inflammation and even complications such as stress tibial fractures could be detected. The total numbers of the patient's radiological lesions were lower at month 3 (n = 7) but were raised at months 6 to 9 because of two new radiologic lesions in the extremities, and were lower again at month 12 (three lesions detectable in WB MRI). Of interest, at months 3, 6 and 12 the patient did not show any clinical detectable lesion. The patient did not show Crohn's disease-associated CARD15 gene variants (R702W, 1007fs, G908R).

Hypophosphatasia was diagnosed in two patients (5%). In these patients clinically affected by CNO we found reduced serum tissue nonspecific alkaline phosphatase activity. No patient had premature loss of teeth, but one patient had a short stature [16].

### Laboratory tests

Laboratory data (Table 2) showed a mean of 7,961 leucocytes/μl (range 4,360 to 17,030/μl), a mean ESR of 16 mm/hour (range 3 to 110 mm/hour, normal <20 mm/hour), a mean C-reactive protein level of 0.7 mg/dl (range 0 to 13.9 mg/dl, normal <0.5 mg/dl), and a mean ferritin level of 36 μg/l (range 3 to 150 μg/l, normal value 2.3 to 63 μg/l). The mean value for leucocytes and ferritin was in the normal range in initial and follow-up diagnostics. Ferritin levels, however, were significantly higher in the initial examination versus follow-ups (ANOVA,*P *< 0.05). C-reactive protein in the initial laboratory data was slightly raised in multifocal CNO (2.5 mg/dl) and in unifocal CNO (1.7 mg/dl), and normalized in the following months (total CNO ANOVA, *P *< 0.05, data of months 1, 3, 6 and 12 versus month 0). The ESR initially was moderately raised in multifocal CNO (32 mm/hour) but not in unifocal CNO (18 mm/hour), and was less in the following follow-ups (total CNO ANOVA, *P *< 0.05, data of months 1, 3, 6 and 12 versus month 0).

**Table 2:** Laboratory features of patients presenting with chronic nonbacterial osteomyelitis

Characteristic	0 months	1 month	3 months	6 months	12 months	Mean (median) in first year
Leukocytes (/μl)	8,324 (8,150)	8,041 (7,770)	8,127 (8,270)	7,734 (7,575)	7,581 (7,430)	7,961 (7,779)
Erythrocyte sedimentation rate (mm/first hour)	28 (24)	15^a ^(11)	11^a ^(10)	13^a ^(10)	12^a ^(10)	28 (10)
C-reactive protein (mg/dl)	2.4 (1.4)	0.1^a ^(0.0)	0.3^a ^(0.0)	0.3^a ^(0.0)	0.2^a ^(0.0)	0.7^a ^(0.0)
Ferritin (μg/l)	54 (37)	35^a ^(32)	31^a ^(27)	29^a ^(26)	31^a ^(22)	36^a ^(27)

Results presented as mean (median). Statistical analysis performed using analysis of variance. ^a^*P *< 0.05 versus month 0.

**Figure 1:**
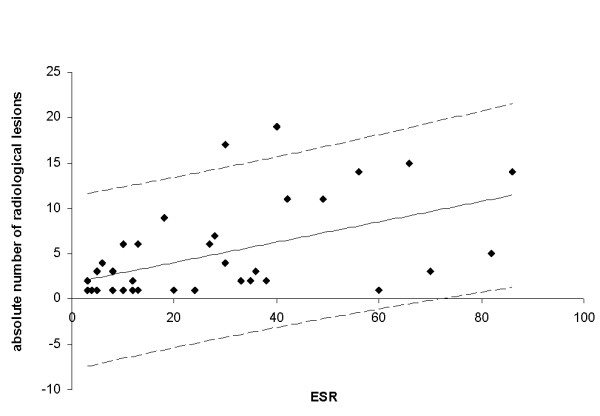
**Correlation of the number of radiological lesions with the erythrocyte sedimentation rate**. Regression line depicts the 95% confidence interval. Results presented as absolute numbers. Correlation coefficient *r *= 0.5, *P *< 0.0009. ESR, erythrocyte sedimentation rate.

As mentioned initially, elevated inflammation markers were noticed in an adolescent with CRMO and acute presentation of inflammatory bowel disease (C-reactive protein, 13.9 mg/dl; ESR, 40 mm/hour; ferritin, 117 μg/l), and in patients with large numbers of radiological inflammatory bone lesions, especially with involvement of the spine or diaphyses of long bones. Patients initially presenting with a higher number of radiological lesions also presented with a higher ESR (*P *< 0.0009, correlation coefficient *r *= 0.5) (Figure 1).

All patients had normal serum IgG levels (mean 1,181 mg/dl) except one patient with CRMO, who initially had raised levels (2,147 mg/dl) that normalized in the following examinations. At diagnosis, serum IgA levels were raised moderately in five cases and raised strongly in one case (mean 186 mg/dl, range 49 to 612 mg/dl) but all showed normal values later on. Serum IgM levels were slightly reduced over time in 10 patients. There was no patient with raised levels (mean 106 mg/dl, range 20 to 285 mg/dl).

Totals of 51.3% and 8.1% of patients had antinuclear antibody (ANA) levels with a titer ≥1:80 and ≥1:160, respectively. These levels of ANA were not different when compared with a healthy control group of 88 age-matched children (data not shown). Eight percent were HLA-B27-positive. No patient was rheumatoid factor IgM-positive. There was no significant difference in the prevalence of ANAs and the presence of RF and HLA-B27 between unifocal CNO and multifocal CNO.

### Imaging techniques

Conventional X-ray scans (only initially) and MRI scans (0, 3, 6, and 12 months) of the region of clinical lesions were performed in all patients. Twenty-one patients (57%) received a WB-MRI at time of diagnosis and after 3, 6 and 12 months of treatment. All together, 475 radiologically defined inflammatory lesions were detected during the first year (initial, 184 lesions; after 3 months, 121 lesions; after 6 months, 89 lesions; after 12 months, 81 lesions), resulting in an overall mean of 3.4 (5.0 at time of diagnosis, 3.7 after 3 months, and 2.5 after 6 and 12 months of treatment) per patient (Table 1). The head was involved in 0.8%, the extremities in 62.7%, the thorax in 9.7%, the spine in 8.4%, and the pelvis in 18.3%. Table 1 shows the course of disease concerning radiological lesions (means) in the first year of treatment.

**Figure 2:**
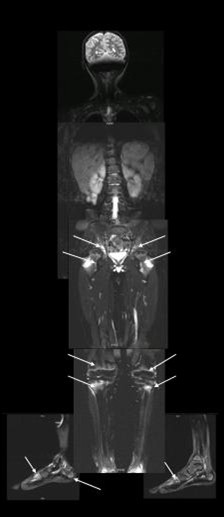
**Whole-body magnetic resonance imaging of chronic nonbacterial osteomyelitis**. Whole-body magnetic resonance imaging of one patient with extensive multifocal inflammatory radiological lesions at time of diagnosis: T2-weighted images with fat suppression (inverse recovery sequences, TIRM). The os sacrum and the acetabulum (all three osseous parts) did show severe signal elevation in the TIRM sequence. Further lesions are seen in the metaphyses of both proximal and distal femurs, proximal tibias and fibulas predominantly in the epiphyses/metaphyses and in the distal tibia and fibula with periosteal edema on the right side, supporting the clinical diagnosis of periostitis and arthritis. Signal alterations/edema in the skeleton of the feet can be noted at the basis of os metatarsale V, os metatarsale I and in the tuber calcanei on the right side; on the left side, the basis and the proximal parts of os metatarsale I and the distal os metatarsale V and the proximal phalanx V are affected.

Most bone lesions of the extremities were localized in the metaphyses of the long bones close to the growth plate - only five patients showed lesions affecting the diaphyses. Inflammatory bone lesions were accompanied with local soft tissue involvement including periosteal, articular and muscular inflammation. Three patients developed pathological bone fractures (spine, two cases; extremities, one case) during the first year. The stress fractures in the distal tibia of both sides in one patient and fractures in the spine of two patients showed a good outcome. Fractures in the extremities did not result in any dislocation, axis deviation or other problems. Patients with spine fractures did not show an active inflammatory process in this region at month 12: one was totally lesion-free, and the other patient showed a lower number of radiological inflammatory bone lesions at month 12. A residual radiological damage could be detected in both patients at month 12 without being symptomatic. Figure 2 shows a WB-MRI with typical inflammatory radiological bone lesions of one patient with CRMO and Crohn's disease at time of diagnosis.

WB-MRI proved to be more sensitive during the study than scintigraphy. Technetium bone scintigraphy has therefore no longer been included in the initial diagnostic work-up after WB-MRI became available. Eleven patients (30%) initially underwent skeletal scintigraphy as WB-MRI had not been available.

### Course of disease

#### Treatment

All patients were treated with naproxen (15 mg/kg/day) for 12 months starting shortly after biopsy. Naproxen was well tolerated during the first year of treatment without any reported adverse events. Naproxen treatment was generally highly effective, inducing a clinical asymptomatic status in 43% (16/37) of our patients after 6 months. Assessment after 6 months revealed progressive disease or no further improvement in four patients. Sulfasalazine in two single doses of 20 mg/kg/day was therefore added, always accompanied by a 2-week course of oral glucocorticoid treatment (prednisone 2 mg/kg/day for 1 week initially, discontinued stepwise afterwards). In one patient with chronic inflammatory bowel disease co-manifestation, a multimodal anti-inflammatory and immunomodulating therapy with naproxen, sulfasalazine and prednisone/budesonid followed by azathioprine was started from the beginning. By using DMARD/glucocorticoid treatment we were able to induce a clinical lesion-free status in two patients, a decreased number of lesions in one patient, and two patients showed an unchanged number of clinical lesions. We did not detect new or more clinical lesions after adding sulfasalazine.

Further on after starting additional treatment with sulfasalazine, the CNO overall disease activity estimated by the parents/patients and by the physician on the VAS and the CHAQ score could be improved (overall disease activity - physician and patient: after 6 months, 1.5; after 12 months, 1; and CHAQ score: after 6 months, 0.1; after 12 months, 0), demonstrating a similar clinical outcome when compared with all patients. The total number of radiological lesions decreased in two patients, persisted unchanged in one patient but increased in two patients.

#### Prognosis

Most children did show a favorable clinical outcome after 1 year of treatment. After 12 months no patient complained about morning stiffness (3 and 6 months, 0/37 patients) or showed a functional impairment of the legs (3 months, 4/37 patients; 6 months, 2/37 patients), and only 14% (5/37 patients) showed local bone/tissue swelling (3 and 6 months, 8/37 patients) and asymmetry of the extremities or thorax (3 months, 11/37 patients; 6 months, 8/37 patients) (Table 3).

**Table 3:** Clinical course of disease 1

Symptom	0 months	1 month	3 months	6 months	12 months	Mean in first year
Morning stiffness	6 (16.2)	1 (2.7)	0 (0)	0 (0)	0 (0)	1.4
Functional impairment of legs	25 (43.2)	14 (37.8)	4 (10.8)	2 (5.4)	1 (0)	9.2
Local bone/tissue swelling	15 (40.5)	14 (37.8)	8 (21.6)	8 (21.6)	5 (13.5)	10.0
Asymmetry of extremities/thorax	14 (37.8)	12 (32.4)	11 (29.7)	8 (21.6)	5 (13.5)	10.0

Results presented as absolute numbers (%). The asymmetry of the thorax was noted predominantly due to rip and sternal involvement. This led to an asymmetric growth and shape of the thorax.

The CNO overall disease activity was initially estimated by the parents/patient as 4.7 on the VAS, after 1 month of treatment as 1.5, after 3 months as 1.1, after 6 months as 0.8, and after 12 months as 0.7 (mean 1.8 in the first year), showing a significant improvement. Severity of disease was initially estimated by the physician as 5.0, after 1 month as 2.4, after 3 months as 1.4, after 6 months as 1.1, and after 12 months as 0.6 (mean 2.1 in the first year), confirming the significant amelioration of complaints.

**Figure 3:**
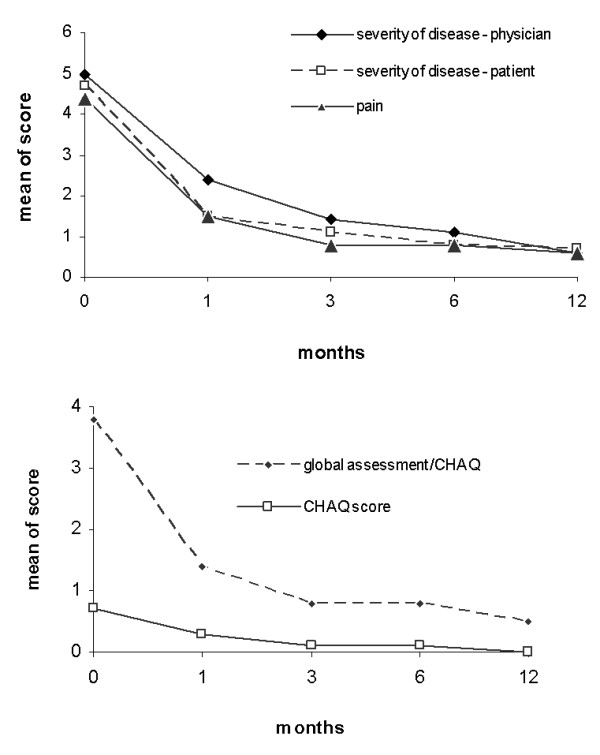
**Clinical course of disease**. Results presented as mean of scores indicated. Statistical analysis performed using analysis of variance. CHAQ, childhood health assessment questionnaire.

**Figure 4:**
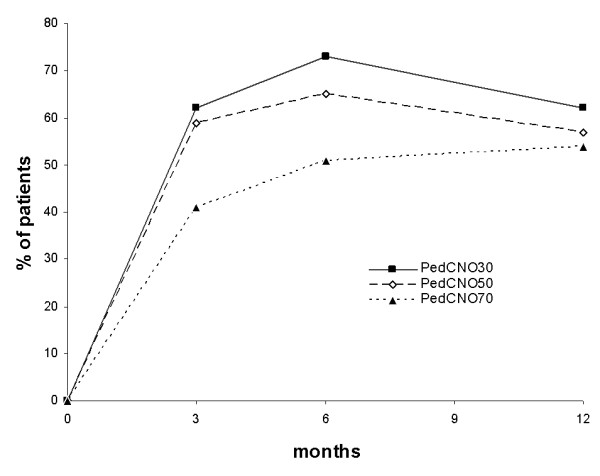
**Course of disease: PedCNO score**. Course of disease with the PedCNO30, PedCNO50 and PedCNO70 scores. Results presented as percentages of the absolute numbers of patients.

Global assessment/CHAQ was initially estimated as 3.8, after 1 month as 1.4, after 3 and 6 months as 0.8, and after 12 months as 0.5 (mean 1.5 in the first year). The CHAQ score was 0.7 at initial presentation, 0.3 after 1 month, 0.1 after 3 and 6 months, and 0.0 after 12 months. Global assessment/CHAQ and the CHAQ score show a significant reduction in the follow-ups (ANOVA, *P *< 0.05) (Figure 3 and Table 4). Patients initially presenting with a higher number of radiological and clinical lesions also presented with a higher CHAQ score (radiological lesion - CHAQ score: *P *< 0.000004, correlation coefficient *r *= 0.6; and clinical lesion - CHAQ score: *P *< 0.0098, correlation coefficient *r *= 0.4). The PedCNO score showed a similar clinical improvement as the single measures (Figure 4). The percentage of PedCNO30 at month 3 was 62%, at month 6 was 72%, and at month 12 was 62%. PedCNO50 responders at month 3 were 59%, at month 6 were 65%, and at month 12 were 57%. The percentage of PedCNO70 responders at month 3 was 41%, at month 6 was 51%, and at month 12 was 54%, respectively.

**Table 4:** Clinical course of disease 2

Symptom	0 months	1 month	3 months	6 months	12 months	Mean in first year
Severity of disease - physician	5.0	2.4^a^	1.4^a^	1.1^a,b^	0.6^a,b,c^	2.1
Severity of disease - patient	4.7	1.5^a^	1.1^a^	0.8^a^	0.7^a,b^	1.8
Pain	4.4	1.5^a^	0.8^a^	0.8^a^	0.6^a^	1.6
Global assessment/CHAQ	3.8	1.4^a^	0.8^a^	0.8^a^	0.5^a^	1.5
CHAQ score	0.7	0.3^a^	0.1^a^	0.1^a,b^	0^a,b^	0.7

Results presented as mean. Statistical analysis performed using analysis of variance. CHAQ, childhood health assessment questionnaire. ^a^*P *< 0.05 versus month 0. ^b^*P *< 0.05 versus month 1. ^c^*P *< 0.05 versus month 2.

#### Course of clinical lesions

After 3 months of treatment 12 patients (32%), after 6 months 16 patients (43%) and after 12 months 23 patients (62%) were clinically lesion free. After 12 months, four patients showed an unchanged number of lesions (one lesion each), three patients showed a lower number (from two to one lesions) and 11 patients had acquired clinical relapses/new lesions during follow-up (partially overlapping cohort). New lesions occurred in one patient at month 1 and in five patients at month 6 as well as month 12. These new lesions involved the extremities in 82% (9/11) of patients. Four of those 11 patients were lesion free at month 12.

#### Course of radiological lesions

Total numbers of radiological lesions were significantly lower at month 6 (n = 89) and month 12 (n = 81) (ANOVA, *P *< 0.05) (Table 1). Concerning the distribution of radiological lesions, only thoracic lesions at month 12 and lesions in the spine and pelvis at months 6 and 12 were significantly less frequent over time (ANOVA, *P *< 0.05). The extremities were the most frequently affected region at all time points. The absolute numbers of lesions in the extremities did decrease; however, relatively the fraction even increased over time (Table 1). The absolute number of lesions in the extremities changed significantly over time (ANOVA for repeated measurements, *P *< 0.05), but does not show a significant difference between the single time points (ANOVA, *P *> 0.05) - most probably due to the small number of patients and the relatively high variance.

After 3 months of treatment one patient (3%), after 6 months five patients (14%) and after 12 months 10 patients (27%) were free of radiological lesions. After 12 months, seven patients (19%) showed an unchanged number of lesions (one to three lesions), five patients (14%) showed a lower number and 15 patients (41%) showed radiological relapses/new radiological lesions at follow-ups. Four new lesions were detected at month 3, five lesions at month 6 and six new lesions at month 12, involving the extremities in 80% (12/15 patients). In patients who showed MRI-defined relapses (n = 15), only one was in radiologic remission before (no radiological lesions at month 6) and only five patients noticed these lesions clinically at the time of the radiological relapse. Of the nine patients initially presenting with radiological lesions in the spine, seven patients were free of active radiological spine lesions after 12 months. Two of the spine lesions were not influenced by treatment.

## Discussion

### Laboratory features

Laboratory parameters were shown to be neither consistent nor entirely predictive for a particular disease course in CNO. Nevertheless, systemic inflammatory markers may be raised to some extent, especially in the multifocal disease type and in patients with associated inflammatory bowel disease. The ESR directly did correlate with the number of radiological lesions, but not clinical lesions. This suggested that the quite often clinically silent lesions can contribute to a measurable systemic inflammation. Of interest, the number of radiologically defined lesions did not strictly correlate with the patient-reported disease activity over time. ANA and HLA-B27 may be present but, according to our patients, neither was the prevalence significantly raised compared with healthy individuals nor were these antigens associated with a more severe course of CNO.

### Scoring the disease

No particular scores have so far been described to measure CNO disease activity. We have used conventional tools established in the analysis of juvenile idiopathic arthritis. The assessment of clinical response in CNO has not so far been standardized. Using a PedCNO score similar to the American College of Rheumatology pediatric score score and the definition of improvement established for juvenile idiopathic arthritis, our results suggest a rapid clinical improvement in our cohort of CNO patients. The lack of specific laboratory markers in children with CNO suggests using the ESR as a biochemical marker of response in the core set. Some children in our cohort have a normal ESR throughout the study, compromising the utility of the definition of improvement. The severity of disease estimated by the patient/parents as a measure to reflect functional impairment or damage and severity of disease estimated by the physician was included based on previous findings in assessment of juvenile idiopathic arthritis. Pain rating was discarded because it is reflected in the severity of disease estimated by the patient/parents. The number of radiological lesions was also included as we consider it of therapeutic relevance.

The CHAQ is the most widely utilized functional status measure in pediatric rheumatology today. The CHAQ has shown to be a valid, reliable, and sensitive functional status measure in children with juvenile idiopathic arthritis [22]. The patient's and the physician's global estimations of disease activity did document the CNO course over time quite comparably, as did the overall pain score (all on a VAS of 0 to 10). It seemed obvious that pain is the most relevant complaint of the patient. The global assessment, as documented in the CHAQ, did report a disease activity comparable with the global severity scores used.

The CHAQ score did correlate directly with the number of radiologically and clinically reported lesions at time of diagnosis. The overall CHAQ score showed the same course over time as the other clinical scores, but had a smaller spectrum of absolute values. In the case of its use in CNO, higher average age and less severe or lack of arthritis probably influences the documented maximum CHAQ score and the validity. Difficulty to perform everyday activity without special aid, devices or assistance was reported rarely, leading to lower or even normal CHAQ levels in comparison with those in juvenile idiopathic arthritis. Some CNO patients may even underestimate their disabilities and discomfort. Therefore, even though improvement was documented in our patients, it remains to be validated whether the CHAQ is a reliable and sensitive functional measure in children with CNO over the long term [23,24].

### Treatment response and prognosis

In our study NSAIDs proved to be an effective therapy concerning clinical aspects (pain, severity of disease, general assessment, CHAQ score, number of clinical lesions) along with a significant amelioration of physical function and quality of life in all patients. Comparable results have been reported before, including our own previous cohort [12,13]. Now we describe in detail the treatment results in the first year showing that 43% of patients can be brought into clinical remission after 6 months and 51% after 12 months of treatment. Radiological absence of lesions (remission) was noted in 14% after 6 months and in 27% of patients after 12 months. Data in the literature are not available for a controlled follow-up in the first 12 months so it is hard to place the treatment response in our patients into a historical comparison. Since no placebo control was used, the overall effectiveness of the treatment can only be estimated. When compared with historical controls, however, an effectiveness seems obvious. Future prospective controlled and randomized trials seem necessary in this regard.

We had chosen to add sulfasalazine as a DMARD after 6 months. Oral glucocorticoids were used as a bridging agent for a limited period of time together with the start of sulfasalazine. By using this strategy we were able to significantly improve the clinical status of the four patients identified as partial responders at month 6.

Nevertheless, it is obvious that the currently preferred treatment is not able to reach remission (radiologically defined) in the majority of patients during/after 1 year. New MRI-defined lesions did even appear in two patients treated with NSAIDs and sulfasalazine from month 6 to month 12. Defining tools to identify patients at risk for a nonresponse to treatment therefore seems of particular relevance.

Additional use of DMARDs already at the time of diagnosis may be helpful and should be considered especially in cases with many inflammatory MRI detectable lesions - as those patients often show a more severe course of disease and may not significantly profit from NSAIDs alone. After 6 months of treatment, clinical and radiological assessment including WB-MRI should be performed in order to assess treatment effects, to detect new inflammatory lesions and progressive disease in order to evaluate therapy escalation. Determining which DMARD might be preferable was not the aim of our study.

In patients with a radiologically lesion-free status (clinical and radiological remission) we did precede according to the protocol as follows: treatment was scheduled for another 6 weeks, and then it was discontinued stepwise. Whether NSAID therapy can be stopped in the long term in case of a radiologically lesion-free (and in our cohort also clinically lesion-free) status still remains to be documented in the further follow-up of our cohort. Furthermore, it is important to note that this was not a placebo-controlled study, so the effects of treatment can only be described and the efficacy estimated. Future prospective controlled and randomized trials seem necessary in this regard.

Summarizing our clinical and therapeutic findings, most children show a favorable clinical outcome in the first year of anti-inflammatory treatment. Inflammatory radiological lesions were still present in 32% (12/37) of patients after 12 months. New lesions appeared in 41% (15/37) of patients during the first year. These lesions mostly were clinically silent, but may become symptomatic in the later course of disease.

### MRI diagnostics

T2-weighted MRI sequences with fat-suppression techniques were demonstrated to be a very sensitive diagnostic tool at the initial and follow-up examinations. Aside from WB-MRI, technetium bone scans can also be helpful in the initial diagnostic setting. Both methods give an estimation of clinically silent CNO lesions; however, WB-MRI may not be available in all institutions.

The region of the extremities was the most frequently affected site at initial presentation and in relapses, and it showed the lowest rate of improvement. Interestingly, the fraction of lesions in the extremities was increasing over time during follow-up, suggesting a more limited response to NSAID treatment compared with other locations.

New MRI-defined lesions (n = 15) did appear during the first year (3 months, 4/15 patients; 6 months, 6/15 patients; 12 months, 5/15 patients) despite anti-inflammatory treatment. Whether the clinically reported complaints or the MRI-defined number of lesions will be the better predictor of the long-term outcome cannot be determined from the 1-year follow-up. In our experience, however, persistence of lesions as defined at 12-month follow-up (by clinical complaints as well as detected radiologically) point towards a long-term chronic disease. Only 33% (5/15) of patients with newly defined radiological relapses clinically did notice these lesions in parallel, raising the question of whether the decision for further treatment should be made mainly by clinical complaints or by radiological data. We have decided to consider these clinically silent lesions of therapeutic relevance. In this regard it seems already obvious that MRI is of higher sensitivity than the clinical experience of the patient. This impression is supported by the fact that all clinically defined and noted lesions did have an MRI correlate. Long-term data in this prospectively followed cohort may provide information about the clinical and radiological outcome and the therapeutic strategies to choose in this regard.

## Conclusions

In summary we found a sustained response after 1 year of anti-inflammatory treatment using NSAIDs, and in cases of insufficient response sulfasalazine plus short-term prednisone was added with positive therapeutic effect. The major findings of our prospective study were a rapid improvement of disease activity, pain and physical function going along with a reduction of predominantly clinical lesions but also radiological lesions. Relapses and new radiological lesions did occur during follow-up, but may not be recognized by the patient. WB-MRI proved to be very sensitive for initial and follow-up diagnostics.

## Abbreviations

ANA: antinuclear antibody; ANOVA: analysis of variance; CHAQ: childhood health assessment questionnaire; CNO: chronic nonbacterial osteomyelitis; CRMO: chronic recurrent multifocal osteomyelitis; DMARD: disease-modifying anti-rheumatic drug; ESR: erythrocyte sedimentation rate; MRI: magnetic resonance imaging; NSAID: nonsteroidal anti-inflammatory drug; PedCNO: pediatric chronic nonbacterial osteomyelitis score; SAPHO: synovitis, acne, pustulosis, hyperostosis and osteitis; TNF: tumor necrosis factor; VAS: visual analog scale; WB: whole body.

## Competing interests

The authors declare that they have no competing interests.

## Authors' contributions

All authors contributed substantially to this work and have read and approved the final manuscript. All listed authors take full responsibility for the manuscript.

## Supplementary Material

Additional file 1 **Further information about the diagnostic procedures mentioned in Subjects and methods**. A word file presenting more detailed information about subjects and methods. Click here for file
