# Supplementary material for: Chronic nonbacterial osteomyelitis in childhood: prospective follow-up during the first year of anti-inflammatory treatment
Source: Arthritis Res Ther. 2010 Apr 30;12(2):R74. doi: 10.1186/ar2992 (PMC2888230; doi:10.1186/ar2992)
Supplement: Additional file 1 — Further information about the diagnostic procedures mentioned in Subjects and methods. A word file presenting more detailed information about subjects and methods. [file ar2992-S1.DOC]

**Supplement**

**Patients:** The patients in this study were recruited into a prospective observational 5 year follow-up study at the children`s hospital, University of Wurzburg. The study was approved by the local ethics committee. Signed informed consent was obtained from the patients’ parents and from adolescent patients. 37 children (24 girls, 13 boys) diagnosed with CNO were included who were newly diagnosed and who had not received any anti-inflammatory or antibiotic therapy. Here we report the follow-up in the first year of anti-inflammatory treatment.

The disease was assessed using a clinical score (see clinical evaluation), initial diagnostic biopsy, laboratory tests and multiple imaging at 0, 3, 6 and 12 months. Children’s age at onset of symptoms ranged from 2.1-13.9 years (mean age 11.0 years). The mean age at diagnosis was 11.5 years (range 2.2-16.9) with a mean delay of diagnosis of 5 months (range 0-36 months). Patients were diagnosed with uni- or multifocal CNO on the basis of clinical signs of osteomyelitis (pain, local swelling, impairment of limb motion) and a diagnostic procedure including standard laboratory data (all patients), x-ray examination (all patients), technetium bone scan (20 patients) and magnetic resonance imaging (MRI; all patients, 21 patients with WB-MRI). In addition, diagnostic bone biopsy, including extensive microbial investigations (standard culture techniques to detect fungi, mycobacteria, aerobic and anaerobic bacteria in all samples as well as eubacterial PCR for molecular detection of bacterial ribosomal RNA) was done in all patients to rule out bacterial osteomyelitis. Treatment with naproxen 15 mg/kg/day started at time of diagnosis/biopsy and continued throughout 12 months. In four children sulfasalzine and short term oral glucocorticoids were added at month 6 of follow-up.

**Tissue biopsy and microbial workup:** Biopsies were performed at the University orthopedic clinic. Bacterial ribosomal DNA analysis by PCR was done in all biopsies using procedures described previously [11, 12]. For detection of eubacteria by universal 16S rDNA amplification, DNA was extracted from biopsy tissue using a QuiaAmp DNA kit (Quiagen). Universal 16S rDNA amplification as done with primers BAK 11w and PC3mod and AmpliTaq Gold Polymerase (Applied Biosystems) as described previously (15, 18). Negative controls were included in all assays, as well as positive controls in which the samples were spiked with DNA to detect the presence of PCR reaction inhibitors.

Diagnostic bone biopsy was performed in all patients. Histology could exclude other predominantly malignant differential diagnoses and showed signs of chronic inflammation and reparative changes of osseous tissue depending on the time of biopsy in disease course (predominant lymphocytic cell infiltration, osteolysis, edema, marrow fibrosis, trabecular osteoid apposition, periostal hyperostosis, sclerosis), as previously described [12, 18]. Extensive microbial workup did not detect any bacteria consistent with the diagnosis of sterile inflammation.

**Clinical evaluation:** History and complete physical examination for each patient was performed noting clinical signs like morning stiffness in minutes, relieving posture, local bone/tissue swelling, pain and asymmetry of extremities, arthritis next to or far from a clinical lesion and total number of all clinical lesions and their allocation to different body regions (head, extremities, thorax, spine and pelvis). Copresentation of other diseases (hypophosphatasia) and involvement of other organs (e.g. skin, gastrointestinal tract) were documented.

Standard laboratory tests included complete and differential blood count, erythrocyte sedimentation rate (ESR), C-reactive protein (CRP), serum ferritin, serum alkaline phosphatase (AP), serum IgG, IgM, IgA and only at diagnosis antinuclear antibodies (ANA), IgM - rheumatoid factor (RF) and the presence of HLA-B27.

Children and their parents also completed questionnaires to evaluate current physical function and quality of life. Physical function was estimated using the childhood health assessment questionnaire CHAQ. The childhood HAQ has been adapted from the Stanford Health Assessment Questionnaire assessing disability, discomfort and pain. The CHAQ includes 30 questions concerning 8 areas of daily life “dressing and grooming”, “arising”, “eating”, “walking”, hygiene”, “reach” and “grip and activities”. The time frame is over the past week. The response modalities range on a 4-point ordinary scale with 0 for “no difficulty”, 1 for “some difficulties”, 2 for “many difficulties”, 3 for “unable to do”. An additional response modality, “not applicable for age”, could be used if a child was too young for a certain activity. Parents were also asked to indicate the use of any aids or devices or if the child needs help from another person for any of these activities. If aids/devices or assistance from another person for one of the 8 CHAQ areas are required for an activity, the score is further corrected to at least 2 points for the corresponding areas. The CHAQ disability index is calculated by adding the scores for each of the categories and dividing the sum by the number of categories answered. This gives a score from 0 (no or minimal physical dysfunction) to a maximum of 3.0 (very severe physical dysfunction). Discomfort is determined by presence of pain and its severity in the past week. Besides the 30 items, the CHAQ includes a patient`s or parent`s global assessment of the overall patients **well-being (global assessment/CHAQ)** in the previous week on a 0 - 10 cm visual analog scale (VAS) (0 = very well; 10 = very poor) and a patient`s or parent`s global assessment of the child`s **pain** in the previous week on a 0 - 10 cm VAS (0 = no pain; 10 = very severe pain).

Furthermore our questionnaire included patient`s/parent`s and physician’s **global assessment of the CNO disease activity** **(severity of disease – patient/parent and physician)** using a comparable VAS at month 0, 1, 3, 6 and 12.

A core set of outcome variables was established in order to achieve a more standardized assessment of clinical response in CNO. According to the juvenile arthritis disease activity score established by the American College of Rheumatology (ACR) the CNO core set is composed of the following 5 measures: 1. ESR, 2. number of radiological lesions, 3. severity of disease estimated by the physician, 4. severity of disease estimated by the patient or parent, 5. CHAQ. The definition of improvement was as follows for the PedCNO30 (PedCNO50, PedCNO70) at least 30% (50%, 70%) improvement in at least 3 out of 5 core set variables, with no more than 1 of the remaining variables deteriorating by more than 30% (50%, 70%).

**Imaging:** X-ray examination of the region of clinical lesions and MRI including whole body MRI were performed at the University Children`s Hospital, Wurzburg and evaluated by two radiologists with long-term experience in inflammatory bone disorders. For MRI data acquisition a Siemens Symphony (1.5 T) MRI has been used with body and surface coils, coronal and sagittal stack, 4-5 mm thickness, TIRM, TSE 1w and post gadolinium-diethylene triamine penta-acetic acid (Gd-DTPA) TSE 1w fat saturation. In older children 2 sessions within 1 week were needed for whole body MRI. MRI analysis was used to assess the number of radiological lesions, distribution, signal strength, size, location (epiphysis, metaphysis, diaphysis) and soft tissue involvement (periostal reaction, joint effusion/synovitis, myositis).

**Treatment:** Uniformly treatment with naproxen (15 mg/kg/day) started at time of diagnosis/biopsy and continued throughout 12 months. In 4 children assessment after 6 months revealed progressive disease or no further improvement using NSAIDs alone. Therefore, sulfasalzine was added at 20 mg/kg/day as a DMARD [14, 19, 20]. In addition, oral glucocorticoids were administered for 1 week at 2 mg prednisone/kg/day, followed by another week of tapering and discontinuation. One patient did receive multimodal anti-inflammatory therapy (naproxen, sulfasalazine, azathioprine, prednisone) from the start due to concomitant severe inflammatory bowel disease (see chapter associated diseases).

**Statistical analysis:** Clinical and laboratory data were analyzed using Microsoft Excel®. Multiple comparisons were done by 2-way ANOVA analysis. Dependence of initial lesion numbers and lesion-free status after 12 months of treatment was analyzed by X^2^-test. For repetitive measurements ANOVA for repeated measurements was used. P values less than 0.05 were considered significant. Regression analysis was used to analyze dependence of ESR and radiological lesions. Correlation is given as Pearson correlation coefficient.
